# Performance and trend for quality of service in a large HIV/AIDS treatment program in Nigeria

**DOI:** 10.1186/s12981-019-0242-2

**Published:** 2019-10-01

**Authors:** Ahmad Aliyu, Samer El-Kamary, Jessica Brown, Bruce Agins, Nicaise Ndembi, Gambo Aliyu, Jibreel Jumare, Babatunde Adelekan, Patrick Dakum, Alash’le Abimiku, Manhattan Charurat

**Affiliations:** 1 Institute of Human Virology, Nigeria, Maina Court, Plot 252 Herbert Macaulay Way, Central Business District, Garki, Abuja, 9396 Nigeria; 2 Department of Epidemiology and Public Health, University Maryland School of Medicine, Baltimore, USA; 3 Institute for Global Health Sciences, University of California San Francisco, San Francisco, USA; 4 Institute of Human Virology, University of Maryland School of Medicine, Baltimore, USA; 5 Institute of Human Virology, Nigeria (IHVN), Abuja, Nigeria; 6 Academy for Health and Development, Ile-Ife, Nigeria; 7 Department of Medicine, University of Maryland School of Medicine, 725 West Lombard Street, Baltimore, Maryland 21201 USA

**Keywords:** Quality, Performance, Indicators, ART, Nigeria

## Abstract

**Background:**

As antiretroviral therapy (ART) programs expand access, there is an increase in burden to a healthcare system. These results are reduced provider-patient contact time and poor programmatic and patient outcomes. Quality management offers providers a standardized approach for addressing the appropriateness of care to be applied in resource-limited settings. This study aimed to determine the trend of performance on HIV/AIDS quality management indicators of health facilities providing ART over a period of 5 years.

**Methods:**

The annual performance scores of quality of care (QoC) indicators of 31 health facilities providing ART was extracted from a database covering a period of 5 years (from October 2008 to September 2012). The data are percentages that indicate scores of each health facility assessed based on compliance to National ART guidelines categorized into several indicator domains. A Chi square statistic for the trend, as well as test for departure from the trend line was determined. The *p* value associated with each indicator provides the significant level for testing an alternative hypothesis that the rate of change over the period considered for that indicator does not equal to zero. The slope of the regression line also gives the magnitude of the rate of change for each indicator by healthcare level across the review period.

**Results:**

Generally, performance trends showed improvement across most indicator domains. The highest improvement occurred for “3 month loss to follow-up” and “1 year no-visit”, with scores declining from 37 to 3%, and 42% to 12% respectively. However, there was a sharp decline in performance between 2010 and 2012 in weight monitoring of patients (p < 0.01), adherence assessment to ARVs (p < 0.01) and hematocrit measurements (p = 0.01). The aggregate rate of change β, as obtained from the slope of the trend line is highly significant (p < 0.01) for all the quality of care indicators considered, whether improving or declining.

**Conclusion:**

Periodic assessment to determine HIV/AIDS quality of care can guide rapid scale-up of services to achieve universal coverage in resource-limited settings. Determining trends to understand patterns is very useful for improving programmatic and patient outcomes.

## Background

Sub-Saharan Africa had a massive scale-up of antiretroviral therapy (ART) coverage from 3.9 million people at the end of 2009 to about 21.7 million as of the end of 2017, accounting for about 70.5% of the global number of people receiving HIV treatment in that year [1]. Nigeria has an estimated 3.6 million infected with HIV as of 2010 with 1.4 million of the infected in need of ART, but only about 350,000 had access to lifesaving drugs. By 2017, 3.1 million are infected and 1.04 million are on life-saving ART. Therefore, it is still is an important target to scale-up ART services and implement a continuous quality improvement (CQI) system for standard service delivery necessary to stem the tide and achieve epidemic control [2–4].

The burden of HIV/AIDS epidemic and limited access to ART facilitated the re-emergence of disease conditions such as pulmonary tuberculosis (PTB), in addition to poor quality of life attributable to health and socio-economic challenges associated with limited access to ART [5, 6]. However, with increasing access to ART and reduction in morbidity and mortality from HIV/AIDs, the average life expectancy in Nigeria rose from 48 years in 2000 to 54 years in 2018 [7].

Standard ART improves the quality of life (QoL) of HIV/AIDS patients [8–10]. However, improvement of QoL following ARV treatment does not depend only on access and availability of the lifesaving drugs; it also depends on the appropriate execution of recommended components of care including prevention and treatment of opportunistic infections, adherence to treatment and routine monitoring of laboratory investigations, and the clinical follow-up of patients [11–14]. HIV/AIDS requires comprehensive health services similar to those needed for cancer, diabetes, and cardiovascular care [15].

The delivery of HIV/AIDS services in Nigeria started in 2002 with no defined guidelines or clinical standards. However, with increasing access to ARV through international programs like the United States President’s Emergency Plan for AIDS Relief (PEPFAR) and Global Fund for AIDS, Tuberculosis, and Malaria (GFATM), clinical practice guidelines are developed to assist care providers in the delivery of standard care needed by HIV/AIDS patients, but compliance from both caregivers and care recipient has become a huge challenge. A study to identify determinants of nonadherence to ART in Southeast Nigeria found 75% of the respondents not fully adhering to their drug regimen [16].

Global HIV/AIDS programs including PEPFAR and GFATM have worked to develop results-based performance indicators to track achievements towards meeting program goals concerning funding. However, program-level indicators remain incomplete, and a carefully selected set of measures assessing the processes of care delivery collected at 6 to 12-month intervals, would provide important data to guide quality improvement efforts for standard care delivery of HIV care services [17, 18].

The introduction and implementation of HIV quality-Nigeria (HIVQUAL-N) was supported by the United States Centers for Disease Control and Prevention (CDC) to promote the delivery of appropriate care and treatment to HIV infected individuals through understanding of the human resource and infrastructure needs, as well as the challenges involved to implement a comprehensive ART program that focuses on adherence to National guidelines for delivery of HIV/AIDS services. This paper reports the application of HIVQUAL, a system developed by New York State Department of Health, AIDS Institute (NYSDOH-AI) to evaluate performance on quality management in one of the largest HIV treatment programs in sub-Saharan Africa (sSA) [19–21].

The objective of this analysis is to determine the performance and trend of quality of care in a large HIV prevention and treatment program in Nigeria.

## Methods

AIDS Care and Treatment in Nigeria (ACTION) program is the largest HIV care and treatment program in Nigeria funded by PEPFAR through the Institute of Human Virology, University of Maryland (IHV-UMD). In the beginning, ACTION program activities, as well as the implementation of HIVQUAL occurred mainly at tertiary health centers where majority of people living with HIV/AIDS (PLWHA) access care and treatment services.

### Quality of care indicators development

The Nigerian HIV quality of care indicators were jointly developed by US CDC, New York State Department of Health AIDS Institute (NYSDoH-AI) supported through PEPFAR/Health Resource Services Administration (HRSA) as HEALTHQUAL, IHV-UMD, Nigeria Federal Ministry of Health (FMoH) and other stakeholders, to monitor health facility-level performance that define optimal patient and program level outcomes.

Data on these indicators are collected annually. For the purpose of this evaluation, selected HIV/AIDS quality indicators (weight monitoring, treatment adherence assessments, anemia screening, liver function tests (LFT), hepatitis B, C screening, and HIV/AIDS care and support assessment, nutritional assessment, no clinic visit within 1 year, loss to follow-up and tuberculosis screening) were considered. The definition of each quality of care indicator is given in Appendix 1.

### Data collection and description

Four years of annual performance scores of quality of HIV/AIDS care indicators were computed from assessments of 28 tertiary and 3 secondary health facilities providing comprehensive HIV/AIDS care and treatment under the ACTION program. The selection of tertiary centers for HIV care by the ACTION program was to ease access for PLWHA. However, ART services were gradually expanded to several secondary and primary health centers to improve access. Annual audit of sampled patients charts are normally randomly selected at each facility using probability proportional to size sampling that is predetermined to give a 90% confidence interval with a maximum error margin of 16% when using the least number of records. Such audit assessments were conducted in 2008, 2009, 2010, and 2012. Due to program transition between UMB-IHV and IHVN, quality audit assessment was not conducted in 2011. To enable trend evaluation and comparison of scores of quality indicators over the years, only health facilities that collected three or more data points were included.

Performance scores were proportions of patients in the audit samples with appropriate care documented based on specific measures of the selected indicators to indicate facility’s compliance with National HIV/AIDS ART guidelines. The numerator of each indicator represents the number of patients’ charts with documented evidence of compliance to requirements for HIV/AIDS clinical management, while the denominator is the proportionate to size total sample.

### Data analysis

The *p*^*trend*^ command of StataIC 13 statistical software was used to determine trends of performance over the review period. *p*^*trend*^ calculates a Chi square statistic for the trend, as well as test for departure from the trend line. Trend analysis assumes a linear relationship with time; it can detect significant variations over time and identify areas for audit investigation to find solutions, but provides little insight into the root causes of variations. Also, trend analysis does not give normal or baseline benchmarks from which performance can be measured and compared over some time.

The p-value associated with each indicator provides the significant level for testing an alternative hypothesis that the rate of change over the period considered for that indicator does not equal to zero. The slope of the regression line also gives the magnitude of the rate of change for each indicator by healthcare level across the review period, which allows for comparison across the different levels of care. The 95% confidence interval (CI) for each of the quality indicators were computed by sites and year.

## Results

The performance scores for the selected quality indicators across all facilities evaluated are summarized in Table 1. The aggregate percentage scores of quality indicators over the period under review indicate a significant improvement in all domains. Better improvements are seen in percentage of 3 months loss to follow-up from 46% [95% CI 43.8–47.4] in 2008 to 12% [95% CI 10.6–12.8] in 2012, and severe reduction of patients spending 1 year without visit: from 31% [95% CI 29.8–32.8] to 3% [95% CI 2.3–5.0] in the same time period. However, there is decrease in performance between 2010 and 2012 in weight monitoring, adherence assessment, and anemia screening. The trend for each indicator is graphically illustrated in Appendix 2.

**Table 1:** Aggregated scores of HIV/AIDS quality indicators of health facilities from 2008 to 2012

Quality indicators	2008		2009		2010		2012	
	%	95% CI	%	95% CI	%	95% CI	%	95% CI
Weight monitoring	76	74.8–78.1	79	77.8–80.7	76	74.5–77.0	48	46.5–48.9
Adherence assessment	49	47.2–51.3	86	84.6–87.2	91	89.6–91.9	75	72.3–77.4
Hematocrit measurement	15	14.0–17.0	31	29.2–32.7	36	34.2–38.0	26	24.5–27.1
Liver function test	14	12.4–15.2	23	21.1–24.4	29	26.8–30.3	28	27.1–29.7
Hepatitis screening	16	15.1–17.4	27	25.3–27.9	44	42.2–45.1	55	53.4–56.7
Care and support assessment	20	18.6–21.1	48	46.2–49.1	74	72.7–76.0	80	78.8–81.5
Nutritional assessment	28	26.4–30.6	81	79.0–82.4	81	79.7–82.9	93	91.3–93.7
1-year no visit	31	29.8–32.8	32	30.4–33.2	37	36.0–38.9	3	2.3–5.0
Lost to follow up	46	43.8–47.4	36	34.8–38.1	42	40.5–43.6	12	10.6–12.8
TB screening			58	56.1–59.7	73	71.7–75.0	81	79.4–82.4

*CI* confidence interval

### Performance comparison of tertiary and secondary health facilities

The baseline performance scores for most of the indicators were lower for secondary health facilities compared to tertiary except for hepatitis B screening, HIV/AIDS care and support assessment, and 1 year without clinical visit as shown in Table 2. Across both tiers of the health care system, quality performance scores improved from baseline in 2008 to 2010. After 2010, there are declines in weight monitoring, adherence assessment, and hematocrit measurements. Compared to tertiary health facilities, the secondary facilities not only improved favorably but also surpass the highly specialized tertiary sites in almost all indicator domains except on HBV screening and hematocrit measurements.

**Table 2:** Percentage scores of HIV/AIDS quality indicators by health facility type from 2008 to 2012

Quality indicators	Facility type	2008		2009		2010		2012	
		%	95% CI	%	95% CI	%	95% CI	%	95% CI
Weight monitoring	3^0^	78	75.8–79.1	79	77.3–80.4	77	74.9–78.2	48	46.4–49.0
	2^0^	65	58.0–70.7	83	77.7–86.7	72	65.7–77.3	48	44.0–52.5
Adherence assessment	3^0^	48	45.6–49.8	86	84.3–87.1	91	89.9–92.3	73	70.8–75.8
	2^0^	68	60.4–74.4	87	82.4–91.2	87	81.5–90.9	92	85.0–95.5
Hematocrit measurement	3^0^	16	14.2–17.3	32	29.7–33.5	37	34.8–388	26	24.7–27.5
	2^0^	12	7.9–17.8	25	19.5–30.8	29	23.2–35.6	22	17.9–26.8
Liver function test	3^0^	15	13.3–16.2	23	21.1–24.5	29	27.1–30.8	28	26.8–29.6
	2^0^	3	1.3–7.3	22	17.2–28.1	25	19.4–31.3	30	25.4–34.7
Hepatitis screening	3^0^	16	14.8–17.2	28	26.3–29.0	46	44.2–47.3	55	53.5–57.0
	2^0^	18	14.2–23.3	17	13.4–20.6	24	20.4–28.6	52	46.5–58.2
Care and support assessment	3^0^	19	18.1–20.6	47	45.7–48.8	76	74.0–77.4	79	78.0–80.9
	2^0^	25	21.1–30.3	51	46.3–55.7	60	54.0–66.5	88	83.6–91.4
Nutritional assessment	3^0^	31	28.6–33.1	82	80.4–83.8	83	81.7–84.8	92	90.4–93.1
	2^0^	5	1.9–8.9	69	62.7–75.0	58	50.4–65.0	100	97.4–1.0
1 year no-visit	3^0^	30	28.6–31.6	31	29.9–32.8	37	35.1–38.1	4	2.5–5.8
	2^0^	43	37.9–48.5	35	30.8–39.8	45	40.8–50.2	1	0.2–5.4
3 months loss to follow-up	3^0^	46	44.2–48.0	36	34.6–38.1	42	40.2–43.5	12	10.9–13.3
	2^0^	40	33.1–46.5	37	31.3–42.6	44	39.4–49.2	7	4.4–11.0
TB Screening	3^0^	–		57	55.3–59.0	77	75.1–78.5	80	78.0–81.1
	2^0^	–		65	59.5–70.7	38	31.7–44.1	96	92.0–97.8

3^0^, tertiary health care facility; 2^0^, secondary health care facility

The aggregated mean change in slope from 2008 to 2012 was higher for care and support assessment: 0.06 (p^trend^ < 0.01); 3 months lost to follow up: 0.05 (p^trend^ < 0.01). However, mean change of slopes for the secondary facilities over the 5 years was higher compared to tertiary facilities for liver function test (0.04 vs. 0.01), nutritional assessment (0.08 vs. 0.4), 1 year no visit (− 0.04 vs. − 0.01) and tuberculosis screening (0.04 vs. 0.02). There is no difference in the mean change in slope between the two tiers for most of the quality indicators evaluated (Table 3).

**Table 3:** Rate of change of (β) HIV/AIDS quality indicators trend for tertiary and secondary health facilities over the 5-year review period

Quality indicators	Rate of change (β) per year			p-value for trend		
	Aggregated	Tertiary	Secondary	Aggregated	Tertiary	Secondary
Weight monitoring	− 0.03	− 0.03	− 0.03	< 0.01	< 0.01	< 0.001
ART adherence assessment	0.02	0.02	0.02	< 0.01	< 0.01	0.13
Hematocrit measurement	0.01	0.01	0.01	< 0.01	0.01	0.23
Liver function test	0.02	0.01	0.04	< 0.01	< 0.01	< 0.01
Hepatitis serology	0.05	0.05	0.05	< 0.01	< 0.01	< 0.01
HIV/AIDS care and support	0.06	0.06	0.06	< 0.01	< 0.01	< 0.01
Nutritional assessment	0.05	0.04	0.08	< 0.01	< 0.01	< 0.01
1 year no visit	− 0.02	− 0.01	− 0.04	< 0.01	< 0.01	< 0.01
3 months loss-to-follow up	− 0.05	− 0.05	− 0.05	< 0.01	< 0.01	< 0.01
Tuberculosis screening	0.02	0.02	0.04	< 0.01	< 0.01	< 0.01

## Discussion

The approach adopted to evaluate HIV/AIDS quality management implementation in this study is the first of its kind in Nigeria. The assessment compared annual performance and trends of quality of services provided by HIV programs at clinic levels over a period of 5 years. To our knowledge, no similar analysis has been conducted in sub-Saharan Africa (sSA). Tertiary health facilities provide specialty services and represent the most specialized level in the healthcare delivery system. Our findings show that even though secondary health facilities may not provide as much high quality of services at the early years of an HIV program, they catch up and even surpass tertiary levels but over time as the program matures,

Improvement across all indicators from baseline in 2008 to 2010 was likely the result of planned activities designed to address deficiencies identified during the first two successive HIVQUAL-N cycles. The HIVQUAL-N project implemented by IHV-UMB suffered a setback in 2011 following funding challenges and transition to a local IHV-Nigeria. The resultant delayed financial and technical support explain the decline in scores for weight monitoring, adherence assessment, and hematocrit monitoring, and only marginal improvement in the other areas in 2012. This is because these activities are conducted at clinic-level where the workload is often enormous. Indicators measured outside the HIV/AIDS clinics demonstrated better improvement over the same period.

The decline in loss to follow-up, especially from a peak of 42% in 2010 in tertiary health facilities, to 12%, corresponds with findings from a longitudinal analysis of risk factors of patient retention and adherence to ARVs where the loss to follow-up rate was reported as 26% [19].

Generally, it is observed that scores for most of the quality indicators are almost at par between tertiary and secondary levels of care from 2008 to 2010. However, by the last review in 2012, the secondary level of healthcare recorded better improvement than the tertiary level in every quality indicator except for anemia screening (hematocrit measurement) and hepatitis serology, where the difference in scores is also marginal. These findings have programmatic implications for decentralizing HIV/AIDS services to more peripheral health centers such as secondary and primary facilities.

Other studies have shown that the quality of care is preserved when decentralization occurs. A study of patients’ demographic and clinical characteristics and level of care associated with loss to follow-up and mortality in adult patients on first-line ART in Nigerian hospitals, also found that retention of patients is better at secondary health facilities compared to tertiary over a period of 36 months [20]. A report from South Africa found that decentralization to lower clinic levels provided greater proximity and acceptability of services leading to faster enrollment of access to ART and better retention with loss to follow-up reduction from 19 to 2% [21]. Another study in Malawi demonstrated the feasibility of district-wide access to ART in a setting with limited resources for health. Expansion and decentralization of HIV/AIDS service-capacity to primary care level, combined with task shifting, resulted in increased access to HIV services with good program outcomes despite staff shortages [22].

The application of HIVQUAL model to measure of HIV/AIDS quality of service appears adoptable in a resource-limited setting and should be expanded to cover not only treatment, care and support services, but also prevention services, especially elimination of mother to child transmission of HIV and community ART services and palliative care. Similarly, performance measurements for quality indicators should be conducted on a regular schedule in order to consolidate and build upon improvements achieved. It has been shown in this study that temporary breach of a schedule pattern as can be caused by a lack of or inadequate funding and technical support, can cause tremendous setbacks and compromise all gains recorded. This strongly underscores the value of continuous measurement as a stimulus to driving improvement in clinics to sustain gains in performance.

## Figures and Tables

**Fig. 1:**
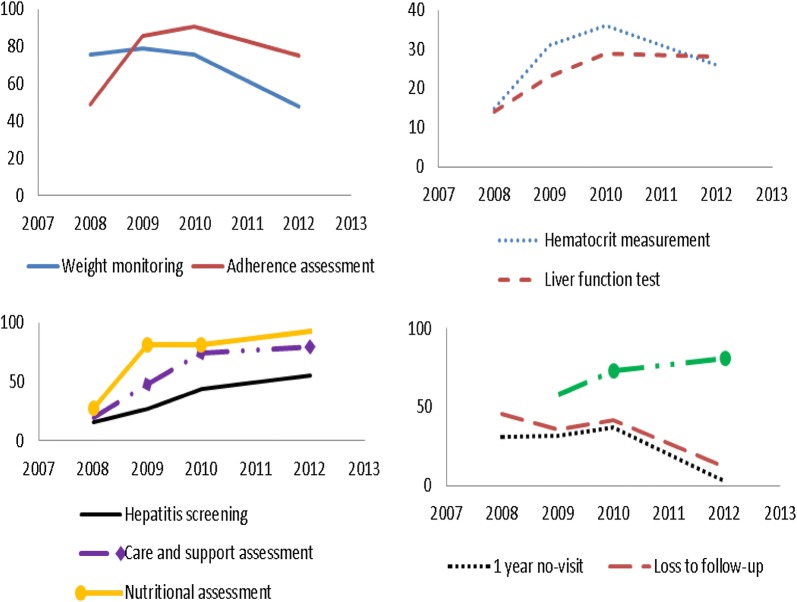
Health facilities performance trends of HIV/AIDS quality indicators in HIVQUAL Nigeria from 2008 to 2012

## Data Availability

The datasets generated and/or analysed in the study are not publicly available due to institutional policy but are available from the corresponding author on reasonable request.

## Appendix 1

See Table 4.

**Table 4:** Quality of care indicators for performance evaluation in HIV Quality Nigeria project

Indicator	Definition
1-year no visit	Proportion of HIV patients who had no visit in the review year (or period)
3 months loss to follow-up	Proportion of patients on ART who had no visit in the last 3 months of the review year (or period)
Liver function test	Proportion of HIV patients on a Nevirapine (NVP)-containing regimen with at least one liver function test (LFT) in the past 12 months
Hematocrit measurement	Proportion of HIV patients on a Zidovudine (ZDV)-containing regimen with at least one hematocrit in the past 6 months
Hepatitis serology	Proportion of HIV patients who have ever had hepatitis serology testing
Tuberculosis screening	Proportion of HIV patients who have been screened for TB during the last 6 months
Weight monitoring	Proportion of HIV patients whose weight is recorded in the past 6 months
Adherence assessments	Proportion of HIV patients on ART who have had an adherence assessment in the last 6 months
Care and support assessment	Proportion of HIV patients who have documented care and support assessment during the review period
Nutritional assessment	Proportion of HIV patients who have documented nutritional assessment in the review period

## Appendix 2

See Fig. 1.

## Abbreviations

ACTION: AIDS Care and Treatment in Nigeria
AIDS: Acquired Immuno-Deficiency Syndrome
ART: anti-retroviral therapy
ARV: anti-retroviral
CI: confidence interval
CQI: continuous quality improvement
FMoH: Federal Ministry of Health
GFTAM: Global Fund for AIDS, Tuberculosis, and Malaria
HIV: human immune-deficiency virus
HIVQUAL: human immune-deficiency virus quality
HIVQUAL-N: human immune-deficiency virus quality-Nigeria
HRSA: Health Resources Services Administration
IHV/UMB: Institute of Human Virology/University of Maryland Baltimore
IHVN: Institute of Human Virology Nigeria
LFT: liver function test
NHREC: National Health Research Ethics Committee
NYSDOH-AI: New York State Department of Health-AIDS Institute
PEPFAR: Presidents’ Emergency Plan for AIDS Relief
SSA: sub-Saharan Africa
p: p-value
PEPFAR: President’s Emergency Plan for AIDS Relief
PLHIV: People living with Human Immunodeficiency Virus
QoC: quality of care
QoL: quality of life
